# An Extract of *Artemisia dracunculus* L. Inhibits Ubiquitin-Proteasome Activity and Preserves Skeletal Muscle Mass in a Murine Model of Diabetes

**DOI:** 10.1371/journal.pone.0057112

**Published:** 2013-02-20

**Authors:** Heather Kirk-Ballard, Zhong Q. Wang, Priyanka Acharya, Xian H. Zhang, Yongmei Yu, Gail Kilroy, David Ribnicky, William T. Cefalu, Z. Elizabeth Floyd

**Affiliations:** 1 Ubiquitin Biology Laboratory, Pennington Biomedical Research Center, Baton Rouge, Louisiana, United States of America; 2 Diabetes and Nutrition Laboratory, Pennington Biomedical Research Center, Baton Rouge, Louisiana, United States of America; 3 Department of Plant Biology and Pathology, Rutgers University, New Brunswick, New Jersey, United States of America; INSERM/UMR 1048, France

## Abstract

Impaired insulin signaling is a key feature of type 2 diabetes and is associated with increased ubiquitin-proteasome-dependent protein degradation in skeletal muscle. An extract of *Artemisia dracunculus* L. (termed PMI5011) improves insulin action by increasing insulin signaling in skeletal muscle. We sought to determine if the effect of PMI5011 on insulin signaling extends to regulation of the ubiquitin-proteasome system. C2C12 myotubes and the KK-A^y^ murine model of type 2 diabetes were used to evaluate the effect of PMI5011 on steady-state levels of ubiquitylation, proteasome activity and expression of Atrogin-1 and MuRF-1, muscle-specific ubiquitin ligases that are upregulated with impaired insulin signaling. Our results show that PMI5011 inhibits proteasome activity and steady-state ubiquitylation levels *in vitro* and *in vivo*. The effect of PMI5011 is mediated by PI3K/Akt signaling and correlates with decreased expression of Atrogin-1 and MuRF-1. Under *in vitro* conditions of hormonal or fatty acid-induced insulin resistance, PMI5011 improves insulin signaling and reduces Atrogin-1 and MuRF-1 protein levels. In the KK-A^y^ murine model of type 2 diabetes, skeletal muscle ubiquitylation and proteasome activity is inhibited and Atrogin-1 and MuRF-1 expression is decreased by PMI5011. PMI5011-mediated changes in the ubiquitin-proteasome system *in vivo* correlate with increased phosphorylation of Akt and FoxO3a and increased myofiber size. The changes in Atrogin-1 and MuRF-1 expression, ubiquitin-proteasome activity and myofiber size modulated by PMI5011 in the presence of insulin resistance indicate the botanical extract PMI5011 may have therapeutic potential in the preservation of muscle mass in type 2 diabetes.

## Introduction

Insulin resistance in clinical states of metabolic syndrome and type 2 diabetes involves multiple tissues, including liver, adipose tissue and skeletal muscle. Specifically, skeletal muscle is the largest contributor to whole-body glucose disposal, making defective insulin signaling in skeletal muscle a primary feature of type 2 diabetes. Along with its role as the primary site of glucose uptake, skeletal muscle is also the main protein reservoir in the body. Protein levels in skeletal muscle are determined by insulin-mediated dual regulation of protein synthesis and protein degradation [1]. Impairment of insulin-stimulated phosphoinositol 3-kinase/Akt signaling is suggested to tilt the balance between protein synthesis and degradation toward protein degradation in skeletal muscle [2], generating amino acids that are released from skeletal muscle to meet whole body energy needs under catabolic conditions. If prolonged, the accelerated protein degradation associated with insulin resistance can lead to loss of skeletal muscle mass and function [3]. A relationship between type 2 diabetes and loss of skeletal muscle mass has been clearly demonstrated in older adults, particularly in women with type 2 diabetes [4] and in sarcopenic muscle loss [5]. Preservation of skeletal muscle mass and strength in this high risk population may depend on strategies designed to diminish the skeletal muscle protein degradation associated with type 2 diabetes.

Protein degradation in skeletal muscle is carried out primarily by the ubiquitin-proteasome system, a complex network of enzymes through which multiple ubiquitin molecules are covalently attached to a protein substrate, leading to degradation of the substrate by the 26S proteasome [6]. Various models of skeletal muscle atrophy show striking increases in components of the ubiquitin proteasome system, particularly the muscle-specific ubiquitin ligases Muscle Atrophy F-box protein (MAFBx, also called Atrogin-1) and Muscle Ring Finger-1 (MuRF-1) [7]. Expression of Atrogin-1 and MuRF-1 [8], [9] as well as proteasome activity [10] is regulated by insulin in skeletal muscle via the PI3Kinase/Akt signaling pathway. The essential role of Atrogin-1 and MuRF-1 in maintaining skeletal muscle mass [11], [12], [13] makes these two muscle-specific ubiquitin ligases attractive targets for pharmacological intervention in insulin resistance and type 2 diabetes.

Botanical extracts have historically been an important source of medically beneficial compounds [14]. Metformin, one of the most commonly used agents in the treatment of type 2 diabetes, was synthesized based on the antihyperglycemic properties of the French Lilac [15].

In this regard, recent studies show that an ethanolic extract of *Artemisia dracunculus* L. (Russian tarragon), termed PMI5011, improves carbohydrate metabolism in animal models of type 2 diabetes [16]. The changes in whole body glucose levels mediated by PMI5011 correlate with increased insulin sensitivity in primary human skeletal muscle cells [17] and in rodent models of type 2 diabetes [18]. PMI5011 enhanced insulin signaling in skeletal muscle is associated with increased phosphatidylinositol 3-kinase activity and Akt phosphorylation along with increased protein content [18]. These results suggest that the effect of PMI5011 in skeletal muscle extends to regulation of ubiquitin-proteasome activity. If so, PMI5011 may be therapeutically useful in the preservation of skeletal muscle mass in insulin resistance and type 2 diabetes.

The aim of this study was to further evaluate the mechanism of action of PMI5011 by determining the effect of PMI5011 on the ubiquitin-proteasome system in skeletal muscle. In particular, we focused on the effect of PMI5011 on ubiquitin-proteasome activity and the expression of Atrogin-1 and MuRF-1 *in vitro* and *in vivo*.

## Materials and Methods

### Ethics statement

This study was carried out in strict adherence to the recommendations in the Guide for the Care and Use of Laboratory Animals of the National Institutes of Health. The animal studies were approved by the Institutional Animal Care and Use Committee of Pennington Biomedical Research Center (protocol number 695).

### Materials

Dulbecco's Modified Eagle's Media (DMEM) was purchased from MediaTech (Manassas, Va). Fetal bovine (FBS) and horse serums were from Hyclone (Logan, UT). The AKT and phospho-AKT (Ser473) antibodies were purchased from Cell Signaling (Danvers, MA), FoxO3a antibody from Millipore (Billerica, MA) and the Atrogin-1 antibody was obtained from ECM Biosciences (Versailles, KY). The MuRF-1 and phospho-FoxO3a antibodies were obtained from Abcam (Cambridge, MA). The IRS-1, PI3K, and 19SRPN2 antibodies were purchased from Upstate (Lake Placid, NY); the ubiquitin antibody from BD Pharmingen (San Diego, CA). All TaqMan primer/probes pairs were obtained from Applied Biosystems (Carlsbad, CA). The 20S Proteasome Activity Assay kit was purchased from Millipore (Billerica, MA) and proteasome substrates were purchased from Boston Biochem (Cambridge, MA) and Bachem (Torrance, CA). Wortmannin, dexamethasone and palmitic acid were obtained from Sigma Aldrich (St. Louis, MO). The Ultra-Sensitive Mouse Insulin ELISA kit was obtained from Crystal Chem (Downers Grove, IL) and the glucose assay kit was from Cayman Chemical (Ann Arbor, MI)

### Sourcing and characterization of PMI5011 extract

The PMI5011 botanical extract from *Artemisia dracunculus* L. was provided by the Botanical Research Center at Pennington Biomedical Research Center. Detailed information about quality control, preparation and biochemical characterization of PMI5011 has been previously reported [14], [16], [17], [18], [19], [20], [21]. PMI5011 was obtained from plants grown hydroponically in greenhouses under uniform and strictly controlled conditions, thereby standardizing the plants for their phytochemical content. The PMI5011 extract was dissolved in DMSO for the *in vitro* experiments.

### Cell culture

Murine C2C12 myoblasts (American Type Culture Collection; Manassas, VA) were cultured in DMEM, high glucose (25 mM) with 10% fetal bovine serum (FBS), 2 mM glutamine, and antibiotics (100 units/ml penicillin G and 100 µg/ml streptomycin). While the myoblasts grew optimally in 25 mM glucose, the glucose concentration was lowered to 5 mM prior to differentiation to minimize any effect of the hyperglycemic conditions on insulin sensitivity of the myotubes. To obtain fully differentiated myotubes, the media was exchanged for DMEM, low glucose (5 mM) with 2% horse serum, glutamine, and penicillin G/streptomycin when the myoblasts reached confluence. The media was replaced every 48 hours and the cells were maintained in this medium. The myotubes were fully formed by the fourth day post-induction.

### Wortmannin treatment

C2C12 myotubes were preincubated with PMI5011 (10 µg/ml) for 16 hours prior to the addition of wortmannin (200 nM). After a 1 hour preincubation in the absence or presence of wortmannin, insulin (100 nM) was added. Whole cell extracts were harvested two hours thereafter for isolation of whole cell extracts. Inhibition of PI3K/Akt signaling by wortmannin was confirmed by loss of Akt phosphorylation.

### Free fatty acid treatment

Palmitic acid was diluted in ethanol (100 mM) and further diluted to a 6 mM working solution in 2% fatty acid free Bovine Serum Albumin (BSA) in DMEM. The 6 mM solution was sonicated and incubated at 55°C until a clear solution was observed. The resulting solution was diluted to the final concentration and filter-sterilized. C2C12 myotubes were incubated in the absence or presence of palmitic acid (200 µM) and PMI5011 (10 µg/ml) for 16 hours in the induction media. Thereafter, the media was exchanged for DMEM containing 0.3% fatty acid free BSA for 6 hours prior to insulin stimulation (100 nM insulin). Two hours after adding insulin, the cells were harvested for isolation of RNA and whole cell extracts.

### Dexamethasone treatment

C2C12 myotubes were incubated in the absence or presence of dexamethasone (1 µM) and PMI5011 (10 µg/ml). When added, PMI5011 was present for 4 hours prior to adding dexamethasone. The cells were harvested for isolation of RNA and whole cell extracts 24 hours after adding dexamethasone.

### Animal studies

KK-A^y^ mice are a murine model of obesity-induced insulin resistance and diabetes causes by mutation of the yellow obese gene A^y^ [22] that was previously used to establish PMI5011 regulates insulin receptor signaling in skeletal muscle [18]. Six-week-old male KK-Ay mice (n = 16) (Jackson Laboratory; Bar Harbor, ME) were single housed in animal rooms maintained at 25°C with a 12-h light–dark cycle. The mice were fed a low-fat diet containing 16.4 kcal% protein, 10.5 kcal% fat, and 71.3 kcal% carbohydrate (D12329; Research Diets, Inc.; New Brunswick, NJ). At 10 weeks of age, the mice were randomly divided into a control group (n = 8) and a PMI5011-treated group (n = 8). The control group was fed the low fat diet *ad libitum* and the PMI5011 treatment group was fed *ad libitum* the low-fat diet containing 1% (w/w) PMI5011. Body weight was recorded weekly and food intake was monitored daily. Fasting glucose and insulin levels were measured at 0, 4, and 8 weeks on the diets.

### Blood glucose and insulin measurements

Serum glucose levels were measured by a colorimetric hexokinase glucose assay and serum insulin levels were assayed via ELISA, according to the manufacturers' instructions. Skeletal muscle (gastrocnemious) was harvested from mice that were fasted for 4 hours prior to euthanasia. Human insulin (Humulin, Eli Lilly, Indianapolis, IN) was administered to a subgroup of the control and PMI5011 mice at a dose of 1.5 U/kg and tissue was harvested after 90 minutes in order to assay potential changes in gene expression while maintaining skeletal muscle in an insulin-stimulated state.

### Histological analysis of skeletal muscle

A portion of the skeletal muscle was fixed in 10% formalin and subjected to standard Hematoxylin and Eosin (H&E) staining. The H&E stained myofibers were scanned (NanoZoomer Digital Pathology, Hamamatsu Corp., Bridgewater, NJ) and the cross-sectional area of the myofibers was calculated from a minimum of fifty myofibers/animal using ImageJ (Research Services Branch, NIH, rsbweb.nih.gov/ij/) software.

### Proteasome activity assay

Proteasome activity in C2C12 cells and skeletal muscle was measured according to the manufacturer's instructions (28) Millipore (Billerica, MA). Briefly, the cell lysates were harvested in 50 mM Tris-Cl, pH 7.4 with 25 mM KCl, 2 mM MgCl_2_, 0.1% Triton X-100, 2 mM ATP, 2 mM PMSF. MgATP is included in the lysis buffer to maintain 26S proteasome activity. Proteasome activity was measured by incubating 20 µg of protein per sample of each lysate at 37°C for 60 min. Chymotrypsin-like activity was assayed with the 7-Amino-4-methylcoumarin (AMC) labeled peptide substrate Suc-Leu-Leu-Val-Tyr-AMC, tryspin-like activity was assayed using Ac-Arg-Leu-Arg-AMC and caspase-like activity using Ac-Nle-Pro-Nle-Asp-AMC. The free AMC released by proteasome activity was quantified using a 380/460 nm filter set (Molecular Devices, Sunnyvale, CA). Proteasome activity is reported as Relative Fluorescence Units (RFU)/µg protein/hr. Each sample was measured in triplicate both in the presence and in the absence of epoxomicin (20 µM, Boston Biochem), a highly specific 26S proteasome inhibitor, to account for any non-proteasomal degradation of the substrate. Non-proteasomal proteolysis is reported as the protease activity occurring in the presence of epoxomicin.

### Analysis of protein expression

Skeletal muscle tissue lysates were prepared by dissecting the muscle free of adipose tissue and homogenizing in 25 mM HEPES, pH 7.4, 1% Igepal CA630, 137 mM NaCl, 1 mM PMSF, 10 µg/ml aprotinin, 1 µg/ml pepstatin, 5 µg/ml leupeptin using a PRO 200 homogenizer (PRO Scientific, Inc., Oxford, CT). The samples were centrifuged at 14,000×*g* for 20 min at 4°C. Whole cell extracts were harvested from the C2C12 myotubes in 50 mM Tris-Cl, pH 7.4 with 150 mM NaCl, 1 mM EDTA, 1% Igepal CA 630, 0.5% Na-deoxycholate, 0.1% SDS, 10 mM N-EM and protease inhibitors, and lysed via sonication. Protein concentrations were determined using a BCA assay (Thermo Fisher Scientific, Rockford, IL) according to the manufacturer's instructions. The tissue supernatants (50 µg) and C2C12 whole cell extracts (50 µg) were resolved by SDS-PAGE and subjected to immunoblotting using chemiluminescence detection (Thermo Fisher Scientific, Rockford, IL) and quantified as described [23].

### Analysis of gene expression

Total RNA was purified from the skeletal muscle tissue using an RNeasy Fibrous Tissue Minikit (Qiagen, Valencia, CA). In each case, RNA (200 ng) was reverse transcribed using Multiscribe Reverse Transcriptase (Applied Biosystems, Carlsbad, CA) with random primers at 37°C for 2 hour. Real-time PCR was performed with TaqMan chemistry using the 7900 Real-Time PCR system and universal cycling conditions (50°C for 2 minutes; 95°C for 10 minutes; 40 cycles of 95°C for 15 seconds and 60°C for 1 minute; followed by 95°C for 15 seconds, 60°C for 15 seconds and 95°C for 15 seconds). The results were normalized to *Cyclophilin B* mRNA or 18S rRNA levels.

### Statistical analysis

Normal distribution of the data for glucose and insulin levels, food intake and body weight was determined using the D'Agostino-Pearson omnibus K2 normality test. Statistical significance for body weight, glucose and insulin levels was determined using two-way mixed model ANOVA with post hoc Bonferroni correction. Statistical significance for all other data was determined using a two-tailed *t* test. All statistical analysis was carried out using GraphPad Prism 5 software (GraphPad Software, La Jolla, CA). Variability is expressed as the mean −/+ standard deviation.

## Results

### PMI5011 regulates expression of Atrogin-1 and MuRF-1 in C2C12 myotubes

**Figure 1:**
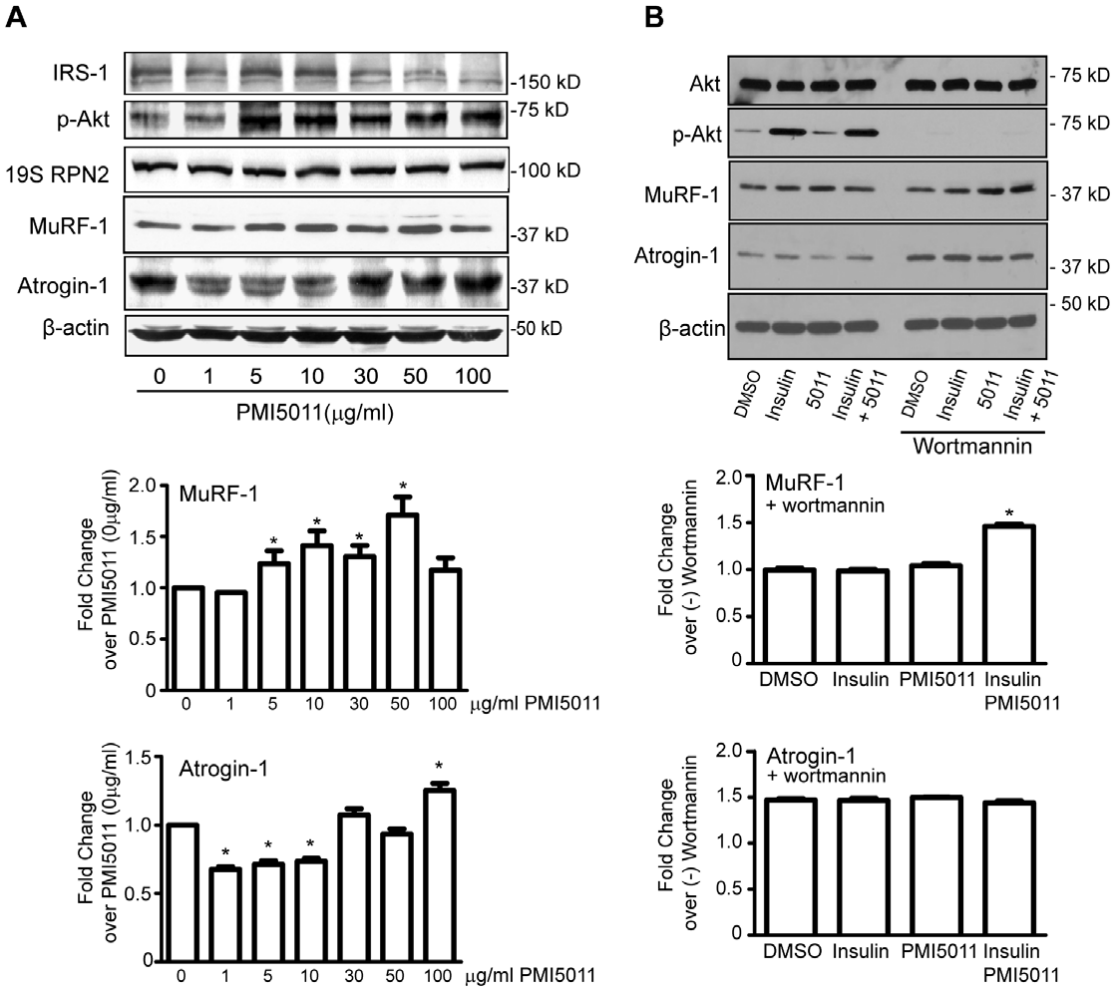
PMI5011 regulates expression of Atrogin-1 and MuRF-1 in skeletal muscle in a PI3K/Akt dependent manner. (A) C2C12 myotubes were incubated with the indicated concentrations of PMI5011 and whole cell extracts were harvested 16 hours thereafter. The levels of IRS-1, pAkt, 19S proteasome subunit RPN2, Atrogin-1 and MuRF-1 were assayed by western blot analysis. The fold change in Atrogin-1 and MuRF-1 expression was analyzed from three separate experiments. The fold change in Atrogin-1 and MuRF-1 protein expression compared to expression in the absence of PMI5011 was analyzed from three independent experiments. (B) C2C12 myotubes were preincubated with PMI5011 (10 µg/ml) for 16 hours as indicated prior to the addition of wortmannin (200 nM). After a 1 hour preincubation with wortmannin, insulin (100 nM) was added as indicated. Whole cell extracts were harvested two hours thereafter and subjected to SDS-PAGE followed by western blot analysis. Inhibition of PI3K/Akt signaling by wortmannin was confirmed by loss of Akt phosphorylation. The fold change in Atrogin-1 and MuRF-1 protein expression in the presence of Wortmannin relative to the corresponding (−) wortmannin conditions was analyzed from three independent experiments. * *p*<0.05.

To determine if the effect of PMI5011 on insulin signaling [18] involves regulating the expression of the Atrogin-1 and MuRF-1 ubiquitin ligases, we assayed Atrogin-1 and MuRF-1 protein expression with increasing amounts of PMI5011 in C2C12 myotubes. As shown in **Figure 1A**, Atrogin-1 protein levels are decreased at concentrations of PMI5011 corresponding to maximal stimulation of Akt phosphorylation while MuRF-1 protein levels show a slight, but significant increase in the presence of PMI5011. Atrogin-1 levels are significantly increased and IRS-1 expression reduced at 100 µg/ml PMI5011, suggesting the beneficial effects of PMI5011 on insulin signaling are limited to 5–10 µg/ml PMI5011 *in vitro*. In contrast, PMI5011 has no effect on the expression of RPN2, a proteasome subunit that is required for funneling substrates into the 20S catalytic core of the 26S proteasome [24].

### The effect of PMI5011 on Atrogin-1 and MuRF-1 expression is mediated by phosphatidylinositol 3-kinase (PI3K) activity

The PI3K/Akt signaling pathway regulates expression of Atrogin-1 and MuRF-1 in skeletal muscle [25], [26]. To determine if PMI5011 regulates Atrogin-1 and MuRF-1 expression via PI3K signaling, a set of experiments was carried out using wortmannin-mediated inhibition of PI3K activity. Inhibition of PI3K activity and Akt phosphorylation increased Atrogin-1 expression in the presence of insulin, PMI5011 or insulin and PMI5011 combined (**Figure 1B**). In contrast, inhibition of PI3K increased MuRF-1 expression only in the presence of insulin and PMI5011 combined when the wortmannin treated MuRF-1 levels were compared to the corresponding untreated samples.

### PMI5011 regulates Atrogin-1 and MuRF-1 expression in hormone-induced insulin resistance

**Figure 2:**
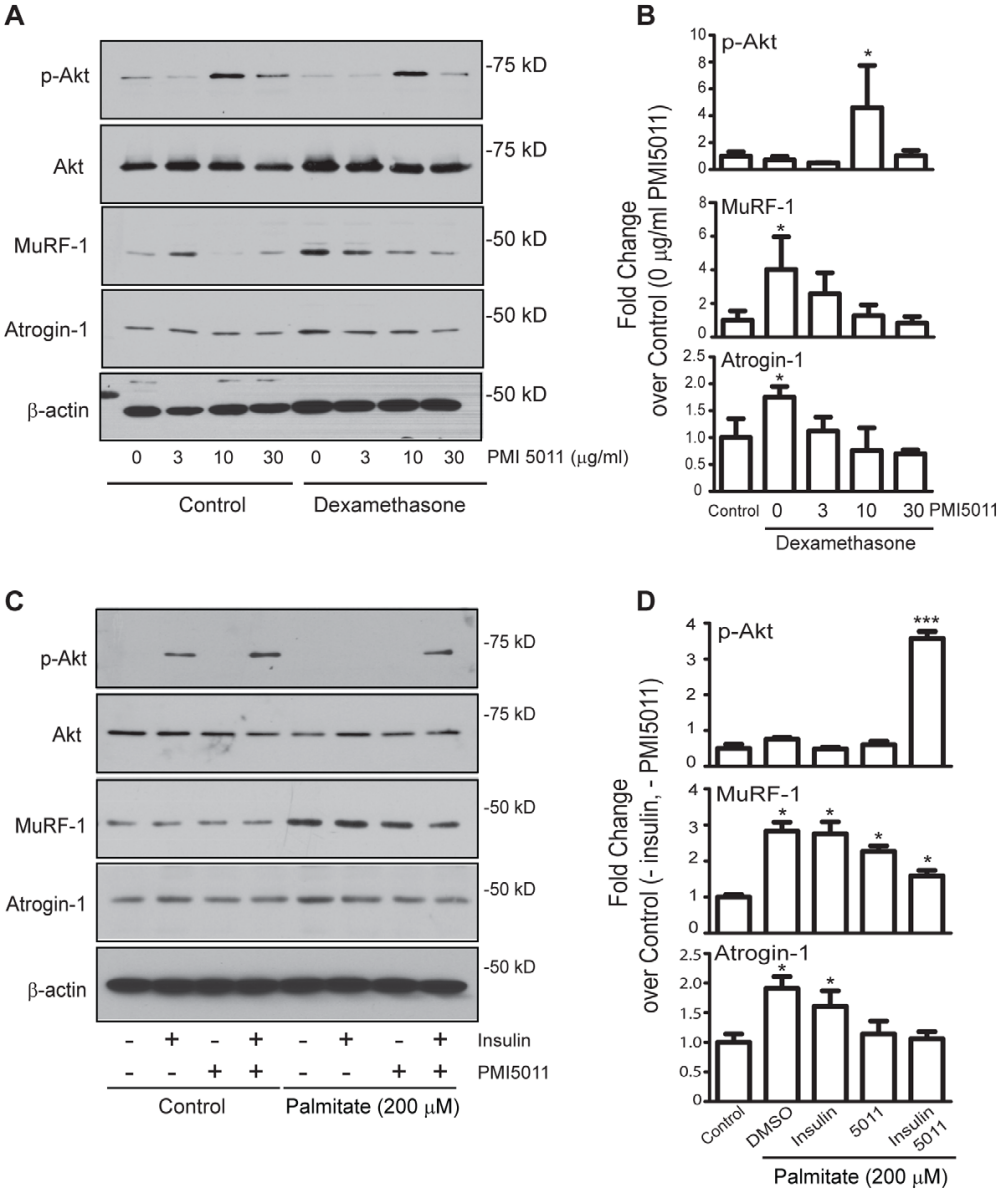
PMI5011 regulates expression of Atrogin-1 and MuRF-1 in two models of insulin resistance *in vitro*. (A) C2C12 myotubes were incubated in the absence (DMSO) or presence of PMI5011 at the indicated concentrations for 16 hours prior to the addition of dexamethasone (1 µM). Twenty-four hour after adding dexamethasone, whole cell extracts were harvested and subjected to SDS-PAGE followed by western blot analysis to determine phospho-Akt, total Akt, Atrogin-1 and MuRF-1 protein levels. β-actin is included as a loading control. (B) The fold change over control for phospho-Akt, MuRF-1 and Atrogin-1 protein levels was analyzed from three independent experiments. * *p*<0.05 compared to control. (C) The C2C12 myotubes were incubated in the absence (DMSO) or PMI5011 (10 µg/ml) for 16 hours prior to adding palmitic acid (200 µM) as indicated. Twenty-four hours later, the cells were serum-deprived for 4 hours prior to insulin-stimulation (100 nM insulin) for 2 hours. Whole cell extracts were harvested and subjected to SDS-PAGE followed by western blot analysis of phospho-Akt, total Akt, Atrogin-1 and MuRF-1 protein levels. (D) The fold change over control for phospho-Akt, MuRF-1 and Atrogin-1 protein levels was analyzed from three independent experiments. * *p*<0.05,*** *p*<0.001 compared to control.

Treatment of C2C12 myotubes with the synthetic glucocorticoid dexamethasone (Dex) induces *atrogin-1* mRNA expression along with other markers of muscle atrophy [27], [28], [29] and inhibits Akt phosphorylation [27], providing an *in vitro* model of hormone-induced insulin resistance and muscle atrophy to assess the effects of PMI5011 in skeletal muscle with impaired insulin signaling. Dex treatment increases Atrogin-1 and MuRF-1 protein levels in the absence of PMI5011 (Figure 2A, see Dexamethasone, 0 µg/ml PMI5011) when compared to the level of each protein in the absence of Dex or PMI5011 (control, 0 µg/ml PMI5011) (**Figure 2 A, B**). However, the Dex-mediated increase in Atrogin-1 and MuRF-1 protein expression is inhibited by PMI5011 (**Figure 2A, B**) with the maximal effect of PMI5011 observed at 10–30 µg/ml. The PMI5011-mediated reduction in Atrogin-1 and MuRF-1 levels coincides with PMI5011 stimulation of Akt phosphorylation in the Dex-treated myotubes (**Figure 2A, B**).

### PMI5011 enhances the effect of insulin on Atrogin-1 and MuRF-1 expression in fatty acid-induced insulin resistance

To determine if PMI5011 regulates Atrogin-1 and MuRF-1 expression in a different *in vitro* model of skeletal muscle insulin resistance, we assayed the effect of PMI5011 on Atrogin-1 and MuRF-1 expression in the presence of palmitic acid, a fatty acid that inhibits insulin signaling in C2C12 myotubes [30], modeling fatty acid induced insulin resistance. To confirm inhibition of insulin signaling by palmitic acid, we assayed the effect of palmitic acid on phosphorylation of Akt (**Figure 2C, D**). As expected, Akt is phosphorylated in response to insulin and the extent of Akt phosphorylation is increased by PMI5011. Palmitic acid inhibits insulin-dependent Akt phosphorylation, but this effect is reversed in the presence of insulin and PMI5011 combined, but not PMI5011 alone. Insulin resistance induced by palmitic acid also modestly increased Atrogin-1 protein expression, but this increase was reversed by the addition of insulin and PMI5011 (**Figure 2C, D**). MuRF-1 protein levels were substantially increased by palmitic acid under all conditions. The palmitic acid-mediated increase in MuRF-1 protein was significantly inhibited when both insulin and PMI5011 were present, but not with the addition of insulin alone (**Figure 2C, D**). Consistent with the results obtained with Dex treatment, the increase in Atrogin-1 and MuRF-1 levels in the presence of palmitate over the levels of each protein in the absence of palmitate (control -insulin, - PMI5011) (Figure 2C,D) is reduced by PMI5011 (Figure 2C,D). Maximal reductions in Atrogin-1 and MuRF-1 expression in the presence of palmitate coincide with an increase in Akt phosphorylation that is mediated by insulin and PMI5011 combined (Figure 2C, D).

### PMI5011 enhances the effect of insulin on proteasome activity and ubiquitylation in C2C12 myotubes

**Figure 3:**
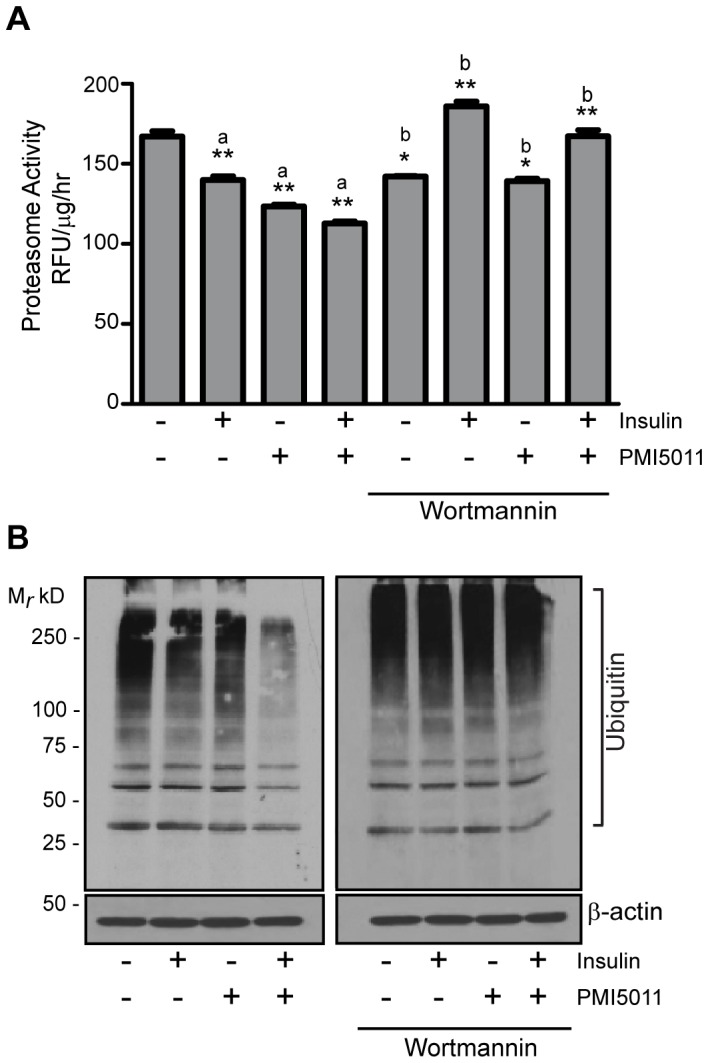
PMI5011 enhances the effect of insulin on proteasome activity and inhibits ubiquitylation in skeletal muscle. C2C12 myotubes were incubated with 10 µg/ml PMI5011 for 16 hours. The cells were subsequently incubated with wortmannin (200 nM) for 1 hour prior to the addition of insulin (100 nM) for 2 hours as indicated. (A) The cells were harvested and assayed for the chymotrysin-like protease activity of the proteasome. Proteasome activity is reported as Relative Fluorescence Units (RFU) RFU/µg protein/hr. The data are reported as the mean −/+ standard deviation from triplicate measurements and are representative of three independent experiments. a = compared to control; b = compared to related treatment (−) wortmannin; *p<0.05, **p<0.01 (B) Whole cell extracts were also subjected to SDS-PAGE followed by western blot analysis using an anti-ubiquitin antibody to assay steady-state ubiquitylation levels. The data are representative of three independent experiments.

We next asked if PMI5011 altered the effect of insulin on proteasome activity and steady-state levels of ubiquitylated proteins *in vitro*. As shown in **Figure 3A**, 26S proteasome activity is significantly decreased in C2C12 myotubes in the presence of insulin or PMI5011 and insulin-mediated modulation of proteasome activity is enhanced by PMI5011. Inhibition of PI3K signaling is associated with increased proteasome activity in the presence of insulin or insulin and PMI5011 combined, indicating PMI5011-mediated enhancement of the effect of insulin on proteasome activity depends on PI3K activity.

We anticipated the decrease in proteasome activity would correlate with an increase in ubiquitylated proteins since the degradation of ubiquitin-modified proteins would be impaired and insulin-mediated changes in proteasome activity are accompanied by accumulation of ubiquitin-conjugated proteins [10]. However, we observed that steady-state levels of ubiquitylation are substantially inhibited in the presence of insulin and PMI5011 combined, but not with PMI5011 alone or in the presence of insulin alone (**Figure 3B**). The effects on ubiquitylation are abrogated in the presence of wortmannin, indicating the changes in steady-state levels of ubiquitylation observed in the presence of insulin and PMI5011 require activation of PI3K.

### PMI5011 regulates ubiquitin-proteasome activity and non-proteasomal protein degradation in skeletal muscle in vivo

**Figure 4:**
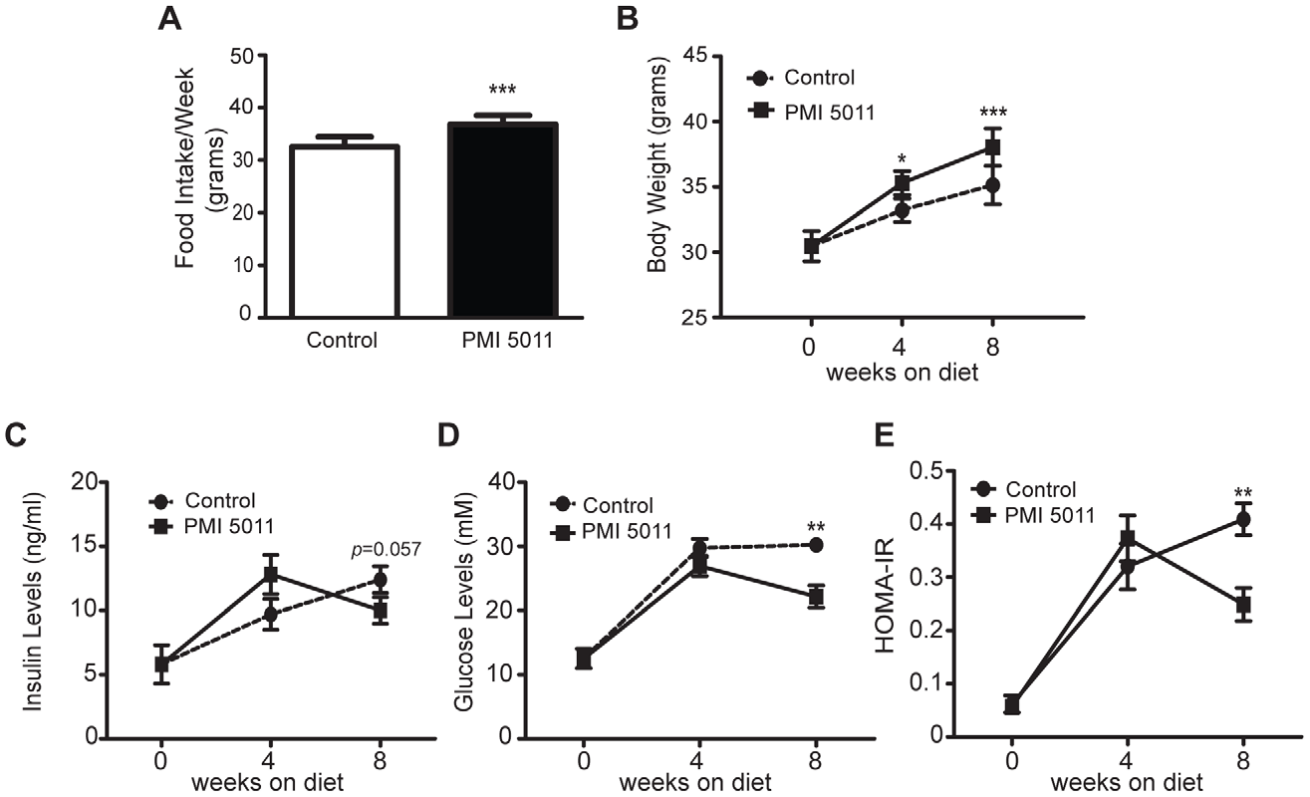
PMI5011 supplementation improves insulin sensitivity *in vivo*. KK-A^y^ mice were singly housed and maintained on a low fat diet (control, N = 8) or a low fat diet containing 1% PMI5011 (w/w) (PMI5011, N = 8) for two months. (A) Food intake was measured daily and (B) body weight has measured each week. (C) Plasma insulin and (D) glucose levels were determined at baseline, 4 and 8 weeks. (E) The index of homeostasis model assessment of insulin resistance [HOMA-IR; insulin (mU/L) x glucose (mM)/22.5] was calculated from fasting glucose and insulin levels.

To determine if our results from the *in vitro* model of fatty acid-induced insulin resistance can be reproduced in an *in vivo* model of insulin resistance, we carried out experiments using the KK-A^y^ model of obesity-related type 2 diabetes. Characterized by severe hyperinsulinemia, hyperglycemia, and hypertriglyceridemia [22], the KK-A^y^ mouse is one of several murine models that show obesity is linked to the development of insulin resistance [22], [31]. Obesity is also associated with an increase in free fatty acids that leads to skeletal muscle insulin resistance [32] and recent evidence indicates that diet-induced obesity leads to skeletal muscle atrophy [33]. To determine if the changes in skeletal muscle protein content reported in the KK-A^y^ murine model of diabetes [18] are accompanied by changes in ubiquitin-proteasome system activity, male KK-A^y^ mice were randomized to PMI5011 dietary supplementation (N = 8 each group) and skeletal muscle was obtained after twelve weeks. The animals treated with PMI5011 had a small, but significant increase in food intake that corresponded with a slight, but significant increase in body weight (**Figure 4A, B**). At the end of eight weeks, serum glucose levels for the PMI5011-fed animals were significantly lower than the control animals and serum insulin levels trended downward (**Figure 4C, D**). These changes indicate improved glucose disposal and are reflected in an improved Homeostatis Model of Assessment of Insulin Resistance (HOMA-IR) (**Figure 4E**), a measure of insulin sensitivity based on the glucose and insulin levels [34].

**Figure 5:**
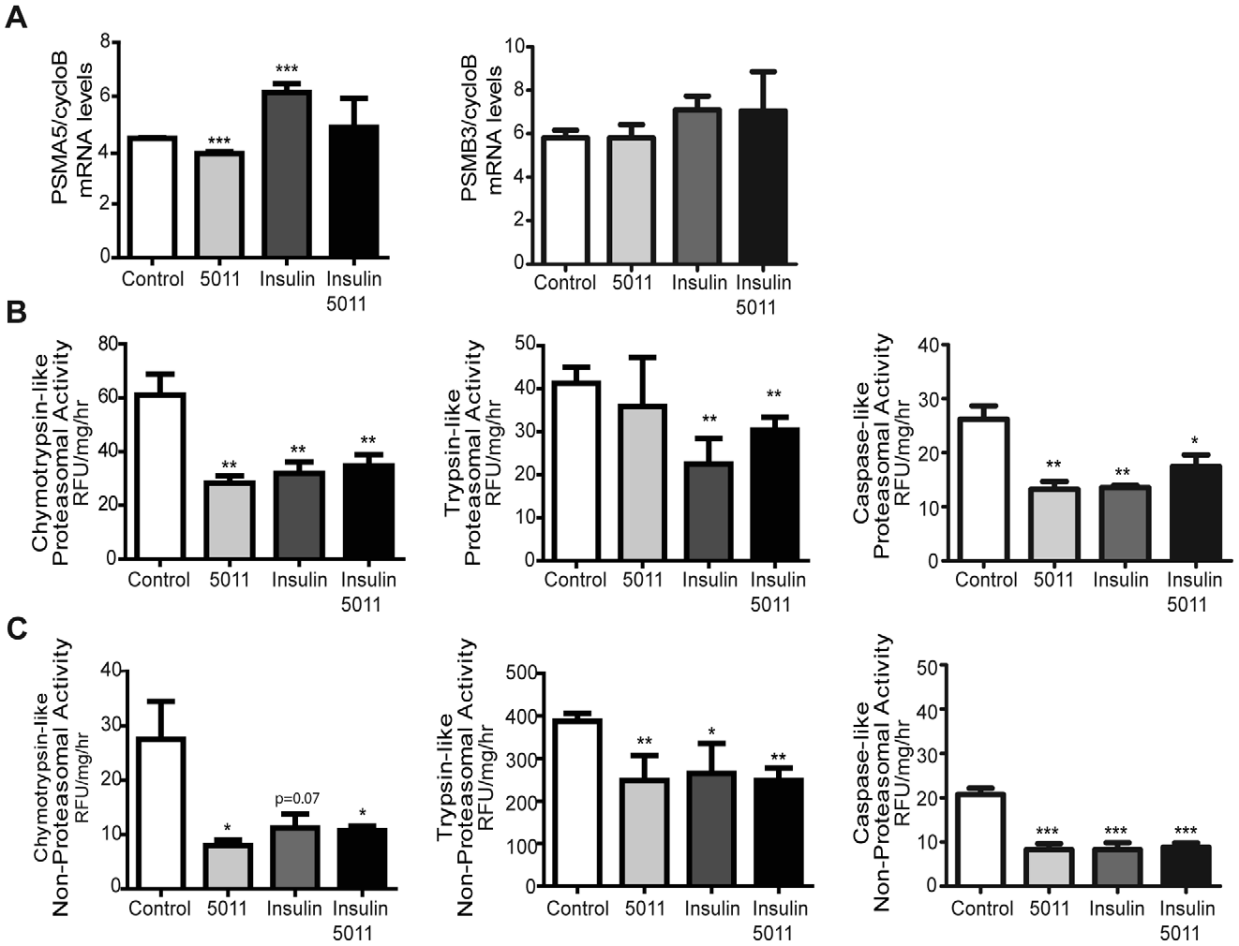
PMI5011 regulates proteasome and non-proteasome protease activity in skeletal muscle. At the end of the study, the KK-A^y^ mice were fasted for 4 hours and insulin (1.5 U/kg) or an equal volume of sterile PBS was administered by intraperitoneal injection to a subgroup (N = 4) of the control or PMI5011 supplemented (N = 4) mice. Skeletal muscle tissue (gastrocnemious) was harvested 90 minutes thereafter. (A) Gene expression of two proteasome subunits, PSMA5 and PSMB3, was analyzed by realtime RT-PCR. (B) The chymotrypin-like, trypsin-like and caspase-like 26S proteasome activities were assayed in a buffer containing MgATP to maintain the 26S proteasome structure. (C) Non-proteasomal protease activity was assayed as the chymotrypsin-like, trypsin-like or caspase-like activity measured in the presence of epoxomicin (20 µM), a highly specific proteasome inhibitor. Proteasome and nonproteasome activities are reported as RFU/µg protein/hr. The data are reported as the mean −/+ standard deviation (4 animals/group). Statistical significance is compared to control. *p<0.05, ** *p*<0.01, ***p<0.001.

Loss of muscle protein in a rat model of diabetes (streptozotocin-induced) is associated with increased expression of genes involved in ubiquitin-proteasome-dependent degradation, including subunits of the 26S proteasome and Atrogin-1 and MuRF-1 [7]. We assayed the effect of PMI5011 on the mRNA levels of two of the 26S proteasome subunits that are strongly upregulated with muscle loss, the 20S proteasome subunits alpha 5 (PSMA5) and beta 3 (PSMB3) [7]. As shown in **Figure 5A**, dietary supplementation with PMI5011 leads to a small, but significant decrease in the gene expression of PSMA5, but not PSMB3. Although decreased expression of PSMA5, with no change in PSMB3 expression suggests specificity in the effect of PMI5011 on proteasome subunit gene expression, PMI5011 had no effect on the gene expression of either proteasomal subunit in the insulin-stimulated muscle. Moreover, the changes in the gene expression of each subunit with acute insulin stimulation do not parallel the changes in proteasome activity, suggesting insulin-mediated regulation of the gene expression of these proteasome subunits does not influence proteasome activity. To determine the effect of PMI5011 on proteasome activity, we assayed the three types of protease activity that constitute proteasome activity (**Figure 5B**): chymotrypsin-like, trypsin-like, and caspase-like activity. PMI5011 substantially reduces chymotrypsin-like and caspase-like proteasome activity without affecting trypsin-like activity. Acute exposure to insulin reduces all three activities, but is not more effective than dietary supplementation with PMI5011 in reducing chymotrypin and caspase-like proteasome activity. PMI5011-mediated changes in non-proteasomal protein degradation mirror the changes observed with chymotrypin and caspase-like proteasome activity (**Figure 5C**). In addition, PMI5011 inhibits the trypsin-like activity of non-proteasome proteases.

**Figure 6:**
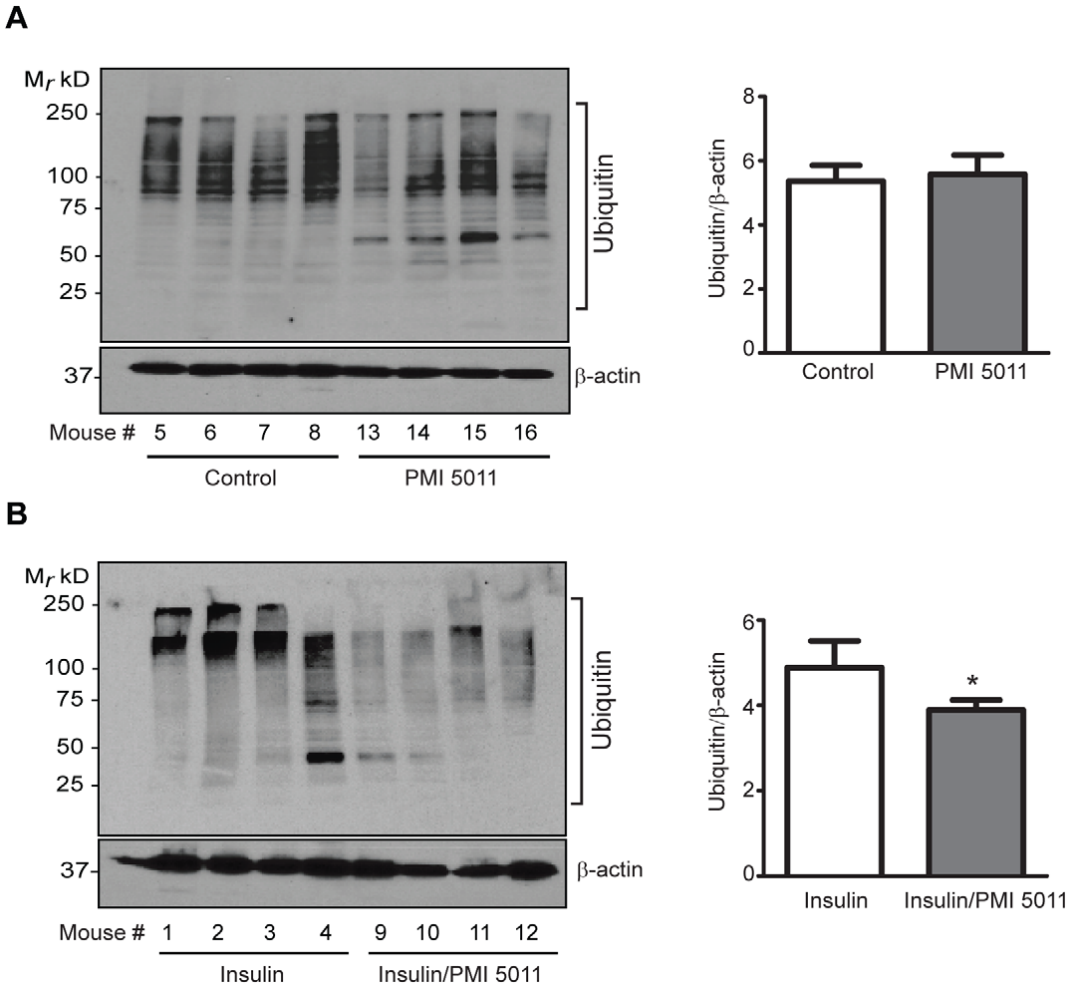
PMI5011 alters ubiquitin conjugation patterns in skeletal muscle. Steady-state ubiquitylation were measured in (A) control (N = 4) and PMI5011 supplemented (N = 4) KK-A^y^ mice or (B) control (N = 4) and PMI5011 supplemented (N = 4) KK-A^y^ mice administered insulin (1.5 U/kg IP) at the end of the study with tissue harvested 90 minutes thereafter. Whole cell extracts were subjected to SDS-PAGE followed by western blot analysis using an anti-ubiquitin antibody. β-actin is included as a loading control. Statistical significance is compared to insulin-treated animals in (B). *p<0.05.

Although proteasome activity is decreased, steady-state levels of high molecular weight ubiquitin conjugates (>75 kD) are significantly (p = 0.031) lower with PMI5011 supplementation compared to the untreated animals while ubiquitin conjugates near 50 kD accumulate with PMI5011, suggesting PMI5011 alters the specificity of proteins modified by ubiquitin without changing the overall level of ubiquitylation (**Figure 6A**). The overall levels of ubiquitylation are lowered by PMI5011 dietary supplementation when compared to insulin, and the pattern of ubiquitin conjugate accumulation also changes (**Figure 6B**), further supporting the notion that PMI5011 alters the specificity of ubiquitin conjugation in skeletal muscle.

### PMI5011 decreases Atrogin-1 and MuRF-1 expression in skeletal muscle in vivo

**Figure 7:**
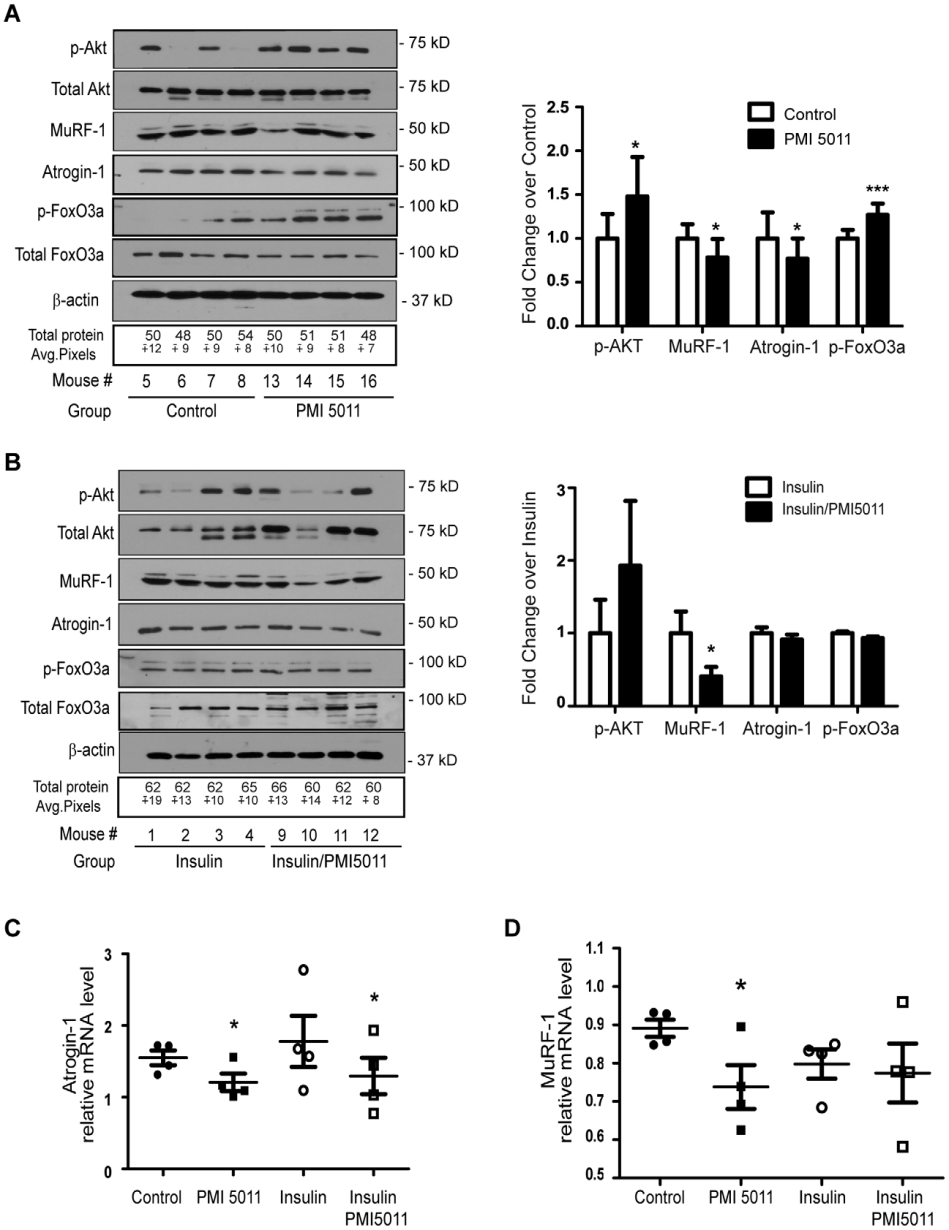
PMI5011 regulates Atrogin-1 and MuRF-1 gene and protein expression in skeletal muscle. (A, B) Skeletal muscle from KK-Ay mice was processed for whole cells extracts and analyzed using SDS-PAGE followed by western blot analysis of phospho-Akt, total Akt, MuRF-1, Atrogin-1, phospho-FoxO3a and total FoxO3a. β-actin and quantitation of the total protein loaded via MemCode staining are included as loading controls. Fold change for phospho-Akt/total Akt, phospho-FoxO3a/total FoxO3a, MuRF-1/total protein and Atrogin-1/total protein is reported for PMI5011 relative to control (A) or PMI5011 combined with insulin relative to insulin alone (B). (C, D) *Atrogin-1* and *MuRF-1* gene expression was determined using realtime RT-PCR. Results are reported as the mean −/+ standard deviation (N = 4/group). * *p*<0.05, *** *p*<0.001. Significance is reported relative to control in (C, D).

PMI5011 supplementation improves insulin signaling in skeletal muscle when assayed as increased phosphorylation of Akt (**Figure 7A**). In addition, PMI5011 enhances insulin-stimulated Akt phosphorylation (**Figure 7B**). The changes in Akt phosphorylation with PMI5011 supplementation correlate with reduced expression of Atrogin-1 and MuRF-1 proteins (**Figure 7A**) while MuRF-1 protein levels are also reduced by PMI5011 when compared to insulin (**Figure 7B**).

The FoxO1and FoxO3a members of the FoxO class of forkhead transcription factors are downstream targets of Akt that regulate the gene expression of *atrogin-1* and *MuRF-1* [25], [27]. To determine if the effect of PMI5011 on atrogin-1 and MuRF-1 protein expression is related to regulation of FoxO phosphorylation, we measured the levels of FoxO3a and phosphorylated FoxO3a (serine 253) in the skeletal muscle. Dietary intake of PMI5011 significantly increases phosphorylation of FoxO3a while the total amount of FoxO3a is decreased compared to the untreated animals (**Figure 7A**). The PMI5011-mediated increase in FoxO3a phosphorylation corresponds to decreased *atrogin-1* and *MuRF-1* expression (**Figure 7C**) when compared to the control animals, consistent with a role for FoxO3a in the effect of PMI5011 on atrogin-1 and MuRF-1 protein levels. Acute insulin treatment does not increase FoxO3a phosphorylation or significantly decrease *atrogin-1* and *MuRF-1* expression in skeletal muscle (**Figure 7B, D**) over that observed with PMI5011 supplementation, although MuRF-1 protein levels are decreased by PMI5011 in the insulin-stimulated muscle (**Figure 7B**).

### Skeletal muscle myofiber size is larger in PMI5011 supplemented animals

**Figure 8:**
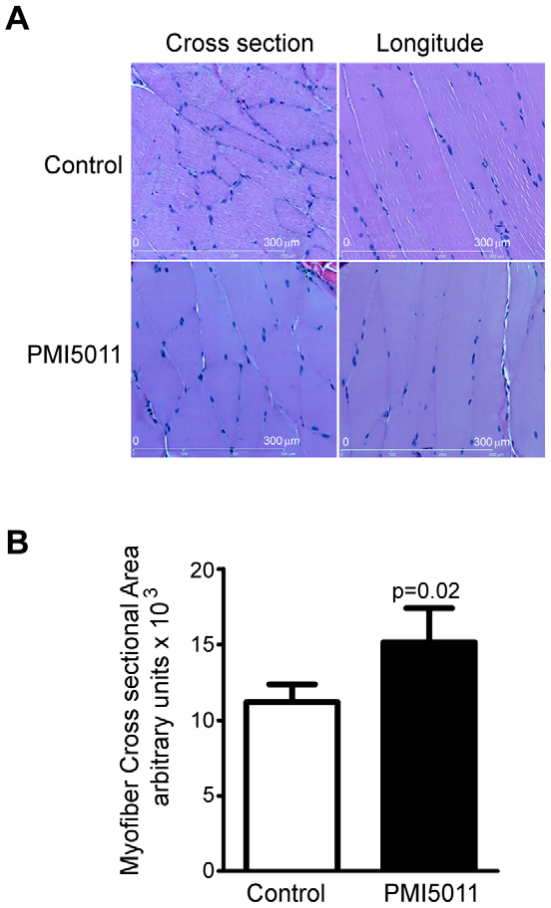
Skeletal muscle myofiber size is larger with dietary intake of PMI5011. (A) H&E staining of cross-section and longitudinal section of gastrocnemious skeletal muscle from control and PMI5011 supplemented KK-Ay mice. (B) The cross-sectional area of fifty myofibers/animal in each group was determined using ImageJ software. The statistical significance is reported as the mean −/+ standard deviation, *p* = 0.02.

Consistent with inhibition of ubiquitylation and proteasome and non-proteasome activity, the cross-sectional area of myofibers from the PMI5011 treated animals was significantly larger than the myofibers from the control animal (**Figure 8A, B**), indicating muscle mass is conserved in the presence of PMI5011.

## Discussion

PMI5011 is a well-characterized botanical extract from *A. dracunculus* L., whose effects on carbohydrate metabolism are comparable to the ability of known antidiabetic drugs (troglitazone and metformin) to lower glucose and insulin levels in murine models of diabetes and insulin resistance [16]. Studies exploring the mechanisms underlying the insulin sensitizing effects of PMI5011 show that PMI5011 enhances insulin signaling in skeletal muscle as demonstrated by increased PI3K activity, increased Akt phosphorylation, and decreased activity of protein tyrosine phosphatase 1B (PTP-1B), which serves as a negative regulator of insulin signaling [18]. The current study provides additional insight into the mechanism of action of PMI5011 in skeletal muscle by demonstrating PMI5011-mediated regulation of the ubiquitin-proteasome system.

Herein, we show PMI5011-enhanced insulin signaling specifically inhibits chymotrypsin-like and caspase-like proteasome activity and all three non-proteasome protease activities, reduces steady-state ubiquitylation levels, regulates expression of the ubiquitin ligases, Atrogin-1 and MuRF-1 and enhances myofiber size in insulin resistant skeletal muscle. In contrast to PMI5011-mediated reductions in Atrogin-1 levels *in vitro* in the absence of insulin resistance (Figure 1A), MuRF-1 levels are increased by PMI5011. PMI5011-mediated decreases in MuRF-1 expression are apparent only in the *in vitro* models of insulin resistance. The PMI5011-mediated change in MuRF-1 expression *in vivo* is comparable to the effect of insulin on MuRF-1 expression. Together, these results suggest MuRF-1 is the more relevant PMI5011 target in the presence of insulin resistance.

PI3K/Akt signaling regulates Atrogin-1 and MuRF-1 protein levels by inhibiting FoxO transcription factor-mediated induction of *atrogin-1* and *MuRF-1* gene expression [25], [26], [27]. Akt-dependent phosphorylation of FoxO1 or FoxO3a excludes the FoxO proteins from the nucleus, either via binding of the phosphorylated FoxO protein to 14-3-3 proteins in the cytoplasm or degradation of the phosphorylated FoxO protein by the proteasome [reviewed in [35]]. The PMI5011-mediated increase in Akt phosphorylation and FoxO3a phosphorylation, coupled with reduced atrogin-1 and MuRF-1 gene expression, suggests PMI5011 mediated reductions in Atrogin-1 and MuRF-1 protein expression are linked to enhanced Akt-dependent regulation of FoxO3a transcriptional activity.

The effects of PMI5011 on Atrogin-1 and MuRF-1 expression are not as pronounced as the PMI5011-mediated inhibition of proteasome and non-proteasome protease activity. As the major site of protein breakdown, proteasome activity is upregulated in muscle loss associated with insulin resistance [2], [3], [7]. PMI5011 substantially reduces the chymotrypsin and caspase-like protease activities in the absence or presence of insulin stimulation. This indicates PMI5011 action has the potential to broadly inhibit protein degradation in insulin resistant skeletal muscle since the proteasomal chymotrypsin-like activity is required for generalized protein degradation, in conjunction with either the trypsin-like or caspase-like activities [36]. However, the proteasome does not degrade intact myofibrillar proteins, the primary group of proteins targeted for breakdown in skeletal muscle atrophy [37], [38]. Although the myofibrillar proteins are ultimately degraded by the proteasome, the filament proteins must be dissociated from the myofibrillar structure for recognition by the proteasome [39]. This task is most likely accomplished by the calpains, calcium-dependent proteases that interact with the ubiquitin-proteasome system [38], [40], although initial cleavage of the filament components may also be carried out by caspases [41]. PMI5011-mediated inhibition of non-proteasome chymotrypsin-like and caspase activities is consistent with an effect of PMI5011 on calpain and caspase proteases activities, suggesting PMI5011 acts to reduce degradation of the myofibrillar proteins by regulating the activity of several classes of proteases.

A potential role for PMI5011 in preventing degradation of the myofibrillar proteins in insulin resistance is further supported by our results showing PMI5011 regulates expression of MuRF-1 in the presence of insulin resistance *in vitro* and *in viv*o. MuRF-1 dependent ubiquitylation of skeletal muscle proteins accounts for the majority of ubiquitin modification in muscle atrophy and MuRF-1directly interacts with and regulates the ubiquitylation of several myofibrillar proteins [42].

A role for MuRF-1 is well established in muscle loss due to insulin resistance associated with fasting or catabolic disease states. But insulin resistance also exacerbates muscle loss associated with aging, termed sarcopenia [43], [44], [45], [46]. In contrast to the rapid muscle atrophy associated with fasting or catabolic diseases, sarcopenic muscle loss occurs gradually and is worsened by obesity [5]. Insulin resistance associated with obesity accelerates sarcopenia by suppressing protein synthesis and stimulating skeletal muscle protein degradation [46], even in the absence of type 2 diabetes [47]. In turn, the loss of muscle mass in sarcopenic obesity increases the risk of developing type 2 diabetes due to decreased glucose disposal in skeletal muscle. There are indications that sarcopenic muscle loss is mechanistically different from rapid muscle loss [48], [49], but enhanced proteasome activity remains a common factor in both forms of muscle loss related to insulin resistance [49]. PMI5011-mediated enhanced insulin signaling, coupled with decreased MuRF-1 expression, decreased 26S proteasome activity and larger myofiber size in the obesity-related insulin resistant animals indicates PMI5011 has therapeutic potential for preserving muscle mass in insulin resistant skeletal muscle, including treatment of muscle loss due to sarcopenia.
